# Comparative Analysis of Virulence Traits and Fluconazole-Response Mechanisms in Clinical Isolates of *Candidozyma auris*

**DOI:** 10.3390/microorganisms14071400

**Published:** 2026-06-24

**Authors:** Cai Hu, Junjie Fang, Hao Zhou, Caiyan Xin, Zhangyong Song

**Affiliations:** 1 School of Basic Medical Sciences, Southwest Medical University, Luzhou 646000, China; hcsypqs@163.com (C.H.); fangjunjiejj@126.com (J.F.); zhjdkl@126.com (H.Z.); 2 Public Center of Experimental Technology, Southwest Medical University, Luzhou 646000, China; 3 Hemodynamics and Medical Engineering Combination Key Laboratory of Luzhou, Luzhou 646000, China

**Keywords:** *Candidozyma auris*, azole resistance, efflux pumps, *ERG11* gene mutation, biofilm formation, virulence factor

## Abstract

*Candidozyma auris* (formerly known as *Candida auris*) has emerged as a formidable clinical fungal pathogen as a result of its multidrug resistance and persistent colonization capabilities. In this study, three clinical *C. auris* strains (namely *C. auris* strain 01, *C. auris* strain 03, and *C. auris* strain 13) with distinct origins were characterized to investigate their phenotypic variations and mechanisms of azole resistance. Comprehensive profiling revealed significant inter-strain differences in biofilm formation, cell surface hydrophobicity, adhesion capacity, and phospholipase activity. Testing for antifungal susceptibility showed that the three clinical strains exhibited different minimum inhibitory concentrations for multiple azoles (fluconazole, voriconazole, and itraconazole) and echinocandins (anidulafungin and micafungin). Sequencing identified Y132F mutations in the *ERG11* gene of the three clinical strains. Mechanistic investigations demonstrated that fluconazole exposure significantly upregulated the expression of efflux pump genes (*CDR1* and *CDR2*) and the genes encoding their transcriptional regulators (*MDR1* and *TAC1b*). In a murine skin colonization model, comparing data from the standard strain *C. auris* strain CBS12766 and clinical strains of *C. auris* strain 03 and *C. auris* strain 13 exhibited a significantly higher fungal burden of tissue, whereas strain *C. auris* strain 01 showed an intermediate level. Host immunity response analysis revealed that expression of the *IL-1β* gene was significantly elevated in *C. auris* strain CBS12766-infected mice, while expression of *IL-6* and *CXCL-1* genes was predominantly increased in the *C. auris* strain 01, with *TNF-α* gene expression levels being comparable across all strains. Histopathological examination confirmed local infiltration of inflammatory cells and mild epidermal edema, indicating active host immune engagement. Overall, our findings highlighted substantial phenotypic heterogeneity, different colonization capacities, and differences in expression of inflammatory cytokines among the *C. auris* strains. Further investigations into fluconazole-response mechanisms identified enhanced efflux pump activity, along with *ERG11* gene Y132F mutations and transcription factor modulation among these clinical strains.

## 1. Introduction

*Candidozyma auris* (formerly *Candida auris*), an emerging multidrug-resistant yeast, has become a serious global health concern since its first identification in 2009 [1]. It is capable of causing a wide range of infections, from superficial colonization of the skin and mucosa to life-threatening bloodstream and systemic infections, particularly in hospitalized and immunocompromised patients [2]. Since 2009, *C. auris* has spread to more than 50 countries, causing outbreaks in intensive care units and long-term care facilities, and leading to disseminated infections with high mortality rates (30–72%) in individuals with underlying conditions or compromised immunity [3]. Notably, *C. auris* demonstrates remarkable environmental persistence and the ability to form stable biofilms on medical surfaces, which facilitates nosocomial transmission and recurrent infections [4]. Previous studies have revealed that its virulence varies considerably among clinical isolates, a phenomenon that may be associated with strain-specific differences in adhesion, biofilm formation, and secreted hydrolytic enzyme activities [5]. However, the specific phenotypic and molecular factors contributing to these differences remain incompletely understood.

Fluconazole, voriconazole, itraconazole, and posaconazole are members of the azole class of antifungal agents and act by inhibiting ergosterol biosynthesis in the fungal cell membrane. Amphotericin B exerts broad-spectrum fungicidal activity by binding to ergosterol and disrupting membrane integrity, whereas 5-fluorocytosine inhibits fungal DNA and RNA synthesis. Another alarming feature of *C. auris* is its extensive content of antifungal resistance genes, especially with respect to azoles such as fluconazole and voriconazole [6]. High resistance rates have been reported worldwide, largely attributable to mutations in the *ERG11* gene, which encodes lanosterol 14α-demethylase, the primary target of azole antifungals [7]. Previous investigations had confirmed that mutations in the target gene *ERG11*, particularly hotspot amino acid substitutions such as Y132F and K143R, are frequently detected in fluconazole-resistant clinical isolates and are strongly correlated with elevated azole minimum inhibitory concentrations [8,9]. In parallel, overexpression of genes encoding drug efflux pumps, including the ATP-binding cassette (ABC) transporter *CDR1* and the major facilitator superfamily (MFS) transporter *MDR1*, contributes to reduced intracellular azole accumulation [10]. Moreover, genes encoding transcriptional regulators such as *TAC1b*, *MRR1*, and *UPC2* have been implicated in azole resistance through the upregulation of *ERG11* and efflux pump genes, further enhancing resistance phenotypes [11,12]. Nevertheless, resistance mechanisms appear to be multifactorial and may also involve transcriptional regulators and stress-response pathways [7]. Despite the increased accessibility to genomic and transcriptomic data, the relationship between molecular resistance determinants and phenotypic virulence traits remains insufficiently explored, particularly among clinical isolates with distinct infection profiles [13].

In the current study, three clinical strains and one standard strain of *C. auris* with differing antifungal susceptibilities and infection capacities were investigated. A series of phenotypic assays, including hydrophobicity, adhesion, biofilm formation, and phospholipase activity, was performed to evaluate key virulence-associated traits. Antifungal susceptibility testing and quantitative analysis of the expression of resistance-related genes (*ERG11*, *CDR1*, *CDR2*, and *MDR1*) were conducted to elucidate fluconazole-response mechanisms. Furthermore, a murine skin colonization model was established to compare in vivo virulence among the different strains through colony-forming unit (CFU) counting, histopathological examination, and quantitative analysis of host inflammatory gene expression. Together, these findings provide insights into the relationship between antifungal resistance and virulence variation in *C. auris*, contributing to a better understanding of its pathogenic potential and toward strategies for infection control.

## 2. Materials and Methods

### 2.1. Reagents, Strains, and Culture Conditions

Amphotericin B, fluconazole, itraconazole, isavuconazole, posaconazole, voriconazole, and 5-fluorocytosine were purchased from Macklin Biochemical Co., Ltd. (Shanghai, China). The standard strain (namely *C. auris* strain CBS12766) was sourced from the American Type Culture Collection (ATCC). The clinical strains used in this study were isolated from patient specimens, confirmed to be *C. auris* in our previous studies, and named *C. auris* strain 01, *C. auris* strain 03, and *C. auris* strain 13 (isolated from blood samples of a patient) [14]. *Candida krusei* 6258 and *Candida parapsilosis* 22019 were obtained from the ATCC and used as quality control strains for antimicrobial susceptibility assays. For experimental procedures, strains were cultured on yeast extract peptone dextrose (YPD) agar medium (containing 2% agar, 2% dextrose, 2% peptone, and 1% yeast extract) for 48 h. Following this primary culture, single colonies were picked out and inoculated into YPD liquid medium, and subjected to aerobic cultivation in a shaking incubator at 200 rpm for 16 h to achieve logarithmic growth. The cell quantification of the corresponding experiment was performed through hemocytometric analysis using an improved Neubauer chamber (QiuJing, Shanghai, China).

### 2.2. PCR Amplification Identification

The specific branch type was determined by comparing the amplification profiles of the various specific primers [15]. PCR reactions were carried out in a total volume of 25 μL containing template DNA, primers, dNTPs, PCR buffer, and DNA polymerase. Amplifications were performed in a thermal cycler under the following conditions: initial denaturation at 98 °C for 30 s, followed by 30 cycles of denaturation at 98 °C for 10 s, annealing of each specific primer at its respective optimal temperature for 25 s, and extension at 72 °C for 30 s, with a final extension at 72 °C for 2 min. PCR products were separated by electrophoresis on a 1% agarose gel stained with a nucleic acid dye and visualized under UV illumination.

### 2.3. Susceptibility to Antifungal Agents

The activities of antifungal agents against all clinical and standard strains were evaluated using the broth microdilution method (Clinical and Laboratory Standards Institute [CLSI] M27-A4) [16]. In brief, each strain was cultured at least twice on antimicrobial-free YPD agar growth media and passaged at 35 °C to ensure purity and viability. The concentration of the activated cells was adjusted to 2.5 × 10^3^ cells/mL. The wells of 96-well plates were loaded with 100 µL of the fungal sample and 100 µL of the diluted antifungal agent, and then the plates were incubated at 35 °C for 48 h. The starting concentrations of the antifungal agents were prepared as follows: Fluconazole, 256 μg/mL; Voriconazole, 256 μg/mL; Itraconazole, 128 μg/mL; Posaconazole, 128 μg/mL; Amphotericin B, 8 μg/mL; and 5-Fluorocytosine, 8 μg/mL. Serial two-fold dilutions were then performed to determine the minimum inhibitory concentrations (MICs). The MIC for amphotericin B was defined as the lowest concentration required to prevent fungal growth. For the azoles and 5-fluorocytosine, the MIC was defined as the lowest concentration required to decrease growth by 50%. The MIC was calculated by measuring absorbance at OD_600_ with a microplate reader.

### 2.4. Adhesion, Biofilm Formation Assay

The adhesion and biofilm formation assays were conducted as previously described, with minor modifications [14]. Fungal strains were initially cultured at 37 °C for 16 h. Cells were then collected by centrifugation at 12,000× *g* for 5 min, washed with phosphate-buffered saline (PBS, pH 7.4), and resuspended in RPMI 1640 medium to a final concentration of 1 × 10^6^ cells/mL. Subsequently, 200 μL aliquots of the suspension were added to 96-well plates and incubated for 4 h at the designated test temperatures to allow the cells to adhere. After incubation, the supernatant was carefully aspirated, and unattached cells were removed by rinsing each well twice with PBS. The remaining adherent cells were then fixed with 100 μL of 10% formaldehyde for 20 min at room temperature. Following fixation, cells were stained with 100 μL of 1 mg/mL crystal violet solution for 30 min. Finally, the wells were gently washed, and the dye was extracted using 100 μL of 95% ethanol (*v*/*v*). Absorbance of the CV solution was measured at 570 nm. The adhesion biomass was calculated as follows: OD_570_ (sample) − OD_570_ (blank), where the blank contained medium and regents without fungal cells. All experiments were performed in three independent replicates. For biofilm formation assays, following the initial adhesion phase, the adherent cells were incubated for an additional 24 h to allow biofilm development. The biofilms were then washed with PBS, fixed with formaldehyde, stained with crystal violet, and quantified as described above.

Biofilm formation can also be assessed by a 2,3-bis-(2-methoxy-4-nitro-5-sulfophenyl)-2H-tetrazolium-5-carboxanilide (XTT) reduction assay, as described previously [14]. Briefly, after the 24 h maturation phase, 100 μL of XTT-menadione working solution was added to each well, followed by incubation in the dark for 2 h at 37 °C. The absorbance of the supernatant was then measured at 450 nm using a microplate reader.

### 2.5. Hydrophobicity Test

The hydrophobicity test was determined as previously described [16]. Briefly, fungal cells were harvested and resuspended in PBS to obtain a cell density of 1 × 10^8^ cells/mL in a final volume of 2.25 mL. The cell suspension was mixed with 0.75 mL of cyclohexane by vortexing for 3 min to allow phase separation. After the suspension was allowed to stand undisturbed for 30 min, a 200 µL aliquot of the aqueous (lower) phase was carefully transferred to a new 96-well plate. The optical density at 570 nm (OD_570nm_) was measured using a microplate reader both before (A_0_) and after (A_1_) 100% cyclohexane treatment. Cell surface hydrophobicity (%) was calculated using the formula (A_0_ − A_1_)/A_0_ × 100, and expressed as percentage hydrophobicity.

### 2.6. Phospholipase Activities

Phospholipase activity was assessed using an egg yolk agar plate assay as previously described [17]. Briefly, 5 μL aliquots of fungal suspension (each containing 5 × 10^6^ cells/mL) were spot-inoculated onto the center of egg yolk agar plates and incubated at appropriate temperatures for 120 h. After incubation, the diameter of the colony and the surrounding precipitation zone (clear ring) were measured. Phospholipase activity was quantified using the Pz value, calculated as the colony diameter divided by the total diameter (colony diameter + precipitation zone diameter). All experiments were performed with three independent replicates for each strain, and the average Pz value was reported.

### 2.7. Structural Prediction and Comparison of ERG11 Proteins

The amino acid sequences of ERG11 proteins were obtained from the NCBI database. Structural models were predicted using the AlphaFold 3 online platform, and for each protein, the model with the highest confidence score was selected for downstream analyses. The predicted structures were visualized and superimposed using PyMOL (version 2.5.5, Schrödinger, LLC, New York, NY, USA). Amino acid substitutions were annotated and mapped onto the three-dimensional structures.

### 2.8. Murine Skin Colonization Models

The protocol of the animal study was approved by the Institutional Animal Care and Use Committee of Southwest Medical University, Luzhou City, Sichuan Province, China (approval 20250227-002). All mice used in this study were female C57BL/6 mice, 6–8 weeks of age, and had a body weight of approximately 20 g. All animals were confirmed to be healthy prior to the initiation of experiments, had not undergone any previous procedures, and were drug-naïve. Experimental groups were assigned randomly. To facilitate colonization, mice were rendered immunocompromised by intraperitoneal injection of cyclophosphamide (100 mg/kg) two days prior to inoculation [18,19]. One day before the fungal challenge, the dorsal hair was removed, and the skin surface was mildly disrupted using a sterile tool to compromise the epidermal barrier. *C. auris* cells used for infection were prepared as described above, washed once with PBS, and resuspended in PBS at a final concentration of 5 × 10^8^ cells/mL. During infection, the inoculum was applied topically to the shaved dorsal skin once daily at the same time for each of three consecutive days. In the control group, an equal volume of PBS was applied to the same area instead of the fungal suspension.

### 2.9. Histopathological Analysis of Mouse Skin Tissues

Mice were humanely euthanized by exposure to 100% carbon dioxide in accordance with institutional guidelines. Skin tissues were obtained from the dorsal region of the murine infection. The samples were excised using sterile scissors and forceps under aseptic conditions and immediately fixed in 10% (*v*/*v*) neutral buffered formalin for 24–48 h at room temperature. After fixation, tissues were processed through graded ethanol dehydration, xylene clearing, and paraffin embedding following standard histological procedures. Paraffin blocks were sectioned at a thickness of 4–5 μm using a rotary microtome, and sections were mounted onto glass slides.

For histological evaluation, sections were deparaffinized and rehydrated, then stained with hematoxylin and eosin (H&E) to assess tissue morphology and inflammatory cell infiltration. Parallel sections were stained with periodic acid–Schiff (PAS) to visualize fungal elements and polysaccharide components within the epidermal and dermal layers. After staining, the slides were dehydrated, cleared, and mounted with neutral resin. Histopathological changes were examined under a light microscope.

### 2.10. Quantification of Fungal Burden in Mouse Skin

To determine the fungal burden in infected skin tissues, mice were euthanized at the indicated time points, and the infected dorsal skin areas were aseptically excised using sterile scissors and forceps. The collected tissues were weighed and placed in sterile microtubes containing 1 mL of sterile PBS. The samples were then homogenized thoroughly using a tissue grinder under aseptic conditions until a uniform suspension was obtained. The resulting homogenates were serially diluted (10^−1^ to 10^−5^) in sterile PBS, and 100 μL of each dilution was spread onto YPD agar plates. Plates were incubated at 37 °C for 24–48 h, and the number of CFU was recorded. The fungal burden was expressed as CFU per g of tissue (CFU/g) by dividing the total CFU count by the corresponding tissue fresh weight.

### 2.11. RNA Extraction and Quantitative Real-Time PCR (qPCR) Analysis

For fungal samples, *C. auris* cells (1 × 10^7^ cells) were cultured in YPD liquid medium and treated with fluconazole at a final concentration of 16 μg/mL for 6 h [20]. Cells were harvested by centrifugation, and total RNA was extracted using a Yeast RNA Extraction Kit (Takara Biotechnology Co., Ltd., Dalian, China) according to the manufacturer’s instructions. RNA purity and concentration were determined spectrophotometrically. Complementary DNA (cDNA) was synthesized from total RNA by reverse transcription using a commercial reverse transcription kit. The qPCR method was performed using TB Green^®^ Premix Ex Taq^TM^ II (Takara Bio Inc. Shiga, Kusatsu, Japan) with gene-specific primers. The *ACT1* gene was used as the internal reference, and relative gene expression levels were calculated using the 2^−ΔΔCt^ method [16]. For infected murine skin samples, dorsal skin tissues were aseptically collected from infected mice at the indicated time points using sterile scissors and forceps. Samples were immediately placed into RNase-free microtubes and snap-frozen in liquid nitrogen. Tissues were homogenized in TRIzol reagent using a tissue grinder until a uniform suspension was obtained. Total RNA was extracted following the manufacturer’s protocol, including DNase I treatment to eliminate genomic DNA contamination. For cDNA synthesis, 1 μg of total RNA from each sample was reverse-transcribed using the PrimeScript^TM^ RT reagent Kit with gDNA Eraser (Takara Bio Inc. Shiga, Kusatsu, Japan). Reverse-transcription quantitative PCR (RT-qPCR) analysis was conducted using SYBR Green Master Mix (Applied Biosystems, Foster City, CA, USA). Expression of host inflammatory (*IL-1β*, *IL-6*, and *TNF-α*) and chemokine *CXCL-1* genes was analyzed, with the mouse *GAPDH* gene serving as the internal control. All reactions were performed with three independent replicates, and relative expression levels were calculated using the 2^−ΔΔCt^ method [16].

### 2.12. Statistical Analysis

All experiments were conducted with three independent replicates. Data were analyzed using GraphPad Prism version 9.0 (GraphPad Software, Inc., San Diego, CA, USA). Differences between two groups were assessed by Student’s *t*-test, while comparisons among three or more groups were evaluated using one-way analysis of variance and Tukey’s pairwise multiple comparison test (ANOVA). In all analyses, a *p*-value of less than or equal to 0.05 was considered to be statistically significant. Data are presented as mean ± standard deviation (SD).

## 3. Results

### 3.1. Biological Characterization of the Four C. auris Strains

**Figure 1:**
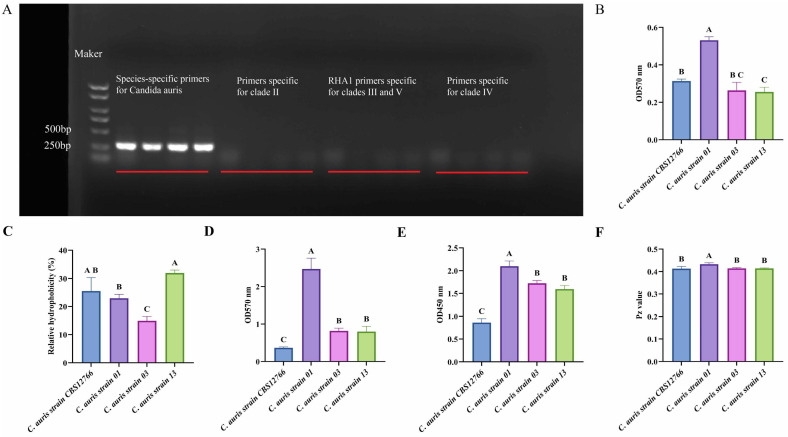
Analysis of clade and virulence characteristics of the four *C. auris* strains. (**A**) PCR amplification using *C. auris*-specific primers generated the expected products in all four isolates. No amplification was observed with clade II-specific primers. Similarly, PCR using *RHA1* primers specific for clades III and V, as well as clade IV-specific primers, produced no detectable products. Collectively, these results indicate that all four isolates belong to clade I. DNA molecular weight was determined using a marker, and the four *C. auris* isolates are shown in the following order: *C. auris* strain CBS12766, *C. auris* strain 01, *C. auris* strain 03, and *C. auris* strain 13. The positions of the electrophoretic bands are indicated by red lines. (**B**) Adhesion ability of the four *C. auris* strains. (**C**) Cell surface hydrophobicity of the four *C. auris* strains. (**D**) Comparison of biofilm formation ability among the four *C. auris* strains, assessed using the crystal violet method. (**E**) Comparison of biofilm formation ability among the four *C. auris* strains, assessed using the XXT method. (**F**) Phospholipase activity of the four *C. auris* strains. Note: Different letters above the bars indicate statistically significant differences following one-way ANOVA and Tukey’s multiple comparison test (*p* < 0.05).

To determine the clade of the four *C. auris* isolates, clade-specific PCR analyses were performed. Species-specific primers produced amplicons of the expected size in all isolates, confirming their identity as *C. auris*. No products were detected using *RHA1* primers specific for clades III and V, or clade II- and IV-specific primers. These results indicate that all four isolates belong to clade I (Figure 1A). Phenotypic variation was assessed in adhesion capacity, hydrophobicity, biofilm formation ability, and phospholipase activity among the four strains. *C. auris* strain 03 and *C. auris* strain 13 had similar biofilm-forming abilities. A similar trend was observed in cellular adhesion, where the adhesion profiles of the four strains closely mirrored their biofilm formation patterns (Figure 1B). For cell surface hydrophobicity, *C. auris* strain 13 exhibited a value similar to that of the standard strain, although differing significantly from those of *C. auris* strain 03 and *C. auris* strain 01 (Figure 1C). Regarding biofilm formation, the *C. auris* strain 01 displayed a significantly greater capacity than that of *C. auris* strain CBS12766, *C. auris* strain 03, and *C. auris* strain 13 (Figure 1D,E); significant differences were also detected between strains *C. auris* strain 03 and *C. auris* strain 13. In terms of phospholipase activity, the *C. auris* strain 01 had significantly different activity from that of the other three strains, while no significant differences were observed among the activities of strains *C. auris* strain CBS12766, *C. auris* strain 03, and *C. auris* strain 13 (Figure 1F).

### 3.2. Comparison of Antifungal Susceptibility and Amino Acid Mutation Analysis of the ERG11 Protein

Antifungal susceptibility testing revealed distinct profiles among the four *C. auris* strains. The *C. auris* strain CBS12766 exhibited MIC values of 32 μg/mL for fluconazole, 0.5 μg/mL for voriconazole, itraconazole, and amphotericin B, and 1 μg/mL for 5-fluorocytosine (Table 1). In contrast, the *C. auris* strain 01 exhibited resistance to fluconazole (MIC > 256 μg/mL) and elevated MICs to voriconazole (32 μg/mL), itraconazole (>64 μg/mL), and posaconazole (>32 μg/mL), while remaining susceptible to amphotericin B (0.5 μg/mL) and 5-fluorocytosine (0.5 μg/mL). The MIC Breakpoints of fluconazole are ≥32 μg/mL, and the MIC Breakpoints of AMB are ≥2 μg/mL [21]. *C. auris* strain 03 and *C. auris* strain 13 showed near-identical susceptibility profiles, with MICs of 4 μg/mL for fluconazole and 1 μg/mL for voriconazole, itraconazole, amphotericin B, and 5-fluorocytosine, respectively (Table 1).

**Figure 2:**
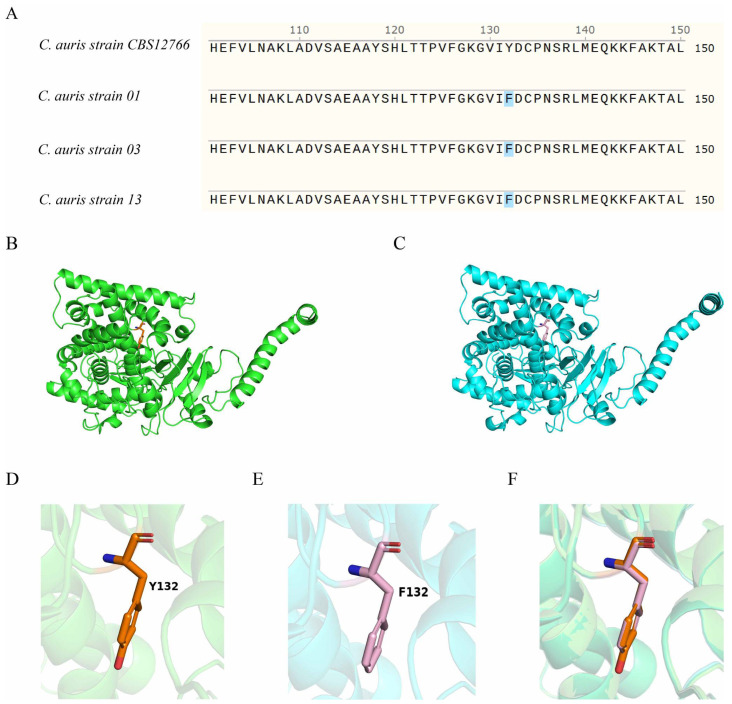
Comparison of antifungal sensitivity and amino acid mutation analysis of the ERG11 protein. (**A**) Multiple sequence alignment of the *ERG11* gene among the four *C. auris* strains. Conserved regions and amino acid substitutions are shown, highlighting sequence variations potentially associated with differences in antifungal sensitivity. Blue “F” marks positions where mutations are observed relative to the reference sequence. (**B**) Spatial position of residue 132 in the ERG11 protein of the reference *C. auris* strain CBS12766. (**C**) Spatial positions of residue 132 in *ERG11* from the three laboratory-isolated *C. auris* strains carrying the Y132F substitution. (**D**) Close-up view of the Y132 residue in the ERG11 protein of the reference *C. auris* strain CBS12766. (**E**) Close-up view of the F132 residue in the ERG11 protein of the three laboratory-isolated *C. auris* strains. (**F**) The Y132 and F132 residues overlap almost perfectly in their 3D backbone structure. Y132 is shown in orange-yellow and F132 in pink to avoid confusion.

The *C. auris ERG11* reference sequence (GenBank accession no. MK059959.1) was retrieved from the NCBI database (*Candidozyma auris* strain CBS 10913 14-alpha-demethylase (*ERG11*) gene-Nucleotide-NCBI, https://www.ncbi.nlm.nih.gov/nuccore/MK059959.1/ accessed on 22 June 2026) and compared with the *ERG11* sequences of the four *C. auris* strains examined in this study. The *ERG11* sequence of the reference *C. auris* strain CBS12766 was identical to that of MK059959.1. In contrast, all three clinical isolates carried the Y132F amino acid substitution. The spatial position of residue 132 in the ERG11 protein of the reference strain is illustrated in Figure 2B, while Figure 2C depicts the corresponding spatial positions of residue 132 in the three clinical isolates. Structural superimposition of the three-dimensional *ERG11* models revealed only subtle conformational differences between the reference and mutant proteins. These structural distinctions are further illustrated in Figure 2D–F. To better illustrate the impact of the Y132F substitution, protein interaction and structural visualization analyses were performed. The Y132F mutation is located within a loop region of *ERG11*. Importantly, neither the wild-type tyrosine nor the mutant phenylalanine at position 132 forms hydrogen bonds, and no hydrogen bond rearrangement was observed following the substitution. The Y132F substitution, therefore, represents a side-chain polarity change, replacing a polar tyrosine with a nonpolar phenylalanine, without altering the hydrogen-bonding network or global protein conformation [22]. Consequently, this mutation is unlikely to affect *ERG11* stability, folding, or macroscopic structure. Its biological impact is instead most likely attributable to the loss of the phosphorylatable phenolic hydroxyl group at position 132. Based on the above-mentioned findings, fluconazole was selected for subsequent mechanistic experiments.

### 3.3. Mechanism of Fluconazole Response in the Four C. auris Strains

**Figure 3:**
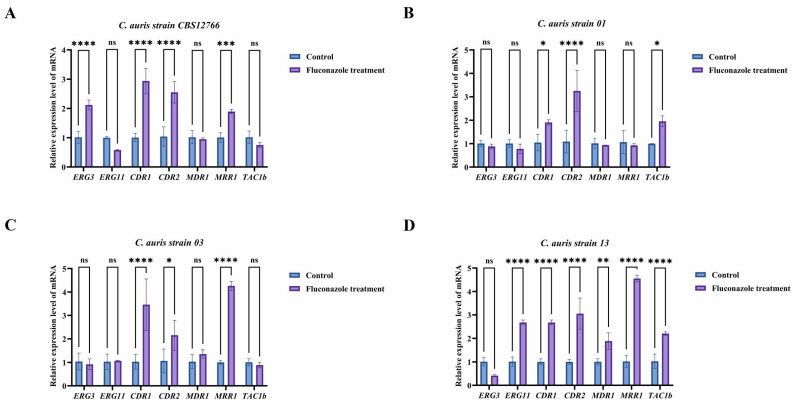
Relative expression of efflux pump genes, transcriptional regulator genes, and key antifungal resistance genes. (**A**) Relative expression of azole-resistance genes in the *C. auris* strain CBS12766. (**B**) Relative expression of azole resistance-associated genes in *C. auris* strain 01. (**C**) Relative expression of azole-resistance genes in *C. auris* strain 03. (**D**) Relative expression of azole-resistance genes in *C. auris* strain 13. Blank control represents samples without drug treatment, whereas fluconazole treatment represents samples treated with 16 µg/mL fluconazole. Data are presented as the mean ± SD of three independent biological replicates. Statistical significance was determined by Student’s *t*-test and denoted as follows: * *p* < 0.05, ** *p* < 0.01, *** *p* < 0.001, **** *p* < 0.0001; ns, not significant.

To investigate the mechanisms underlying response to fluconazole exposure in the four *C. auris* strains, both efflux pump-associated genes (*CDR1*, *CDR2*, and *MDR1*) and the key ergosterol biosynthesis gene *ERG11* were quantitatively analyzed to assess their contribution to azole resistance. The results showed that treatment with 16 μg/mL fluconazole significantly upregulated the expression of the major efflux pump genes *CDR1*, *CDR2*, and *MDR1* (Figure 3). In parallel, the expression of several key azole resistance-associated genes, including *ERG3*, *ERG11*, and *TAC1b*, was also markedly increased in response to fluconazole exposure. However, the fluconazole-response mechanisms differed markedly among the four *C. auris* strains. Distinct resistance-associated transcriptional profiles were observed among the different *C. auris* strains. In the *C. auris* CBS12766 strain, *ERG3*, *CDR1*, *CDR2*, and *MRR1* were significantly upregulated, whereas *MDR1*, *ERG11*, and *TAC1B* remained unchanged, indicating that resistance in this strain is primarily associated with altered sterol biosynthesis and efflux pump regulation (Figure 3A). In contrast, the *C. auris* strain 01 displayed a resistance signature characterized by significant upregulation of *CDR1*, *CDR2*, and *TAC1B*, while *ERG3*, *ERG11*, *MDR1*, and *MRR1* genes showed no detectable changes, suggesting a predominantly *TAC1b*-driven efflux-mediated mechanism (Figure 3B). Similarly, in *C. auris* strain 03, expression of the *CDR1* and *CDR2* genes was significantly upregulated, accompanied by *MRR1* gene upregulation, whereas *ERG3*, *ERG11*, *MDR1*, and *TAC1B* genes were not differentially expressed, indicating a combined contribution of genes controlling efflux pumps and *MRR1*-mediated regulation to resistance (Figure 3C). Notably, *C. auris* strain 13 exhibited a broader resistance-associated transcriptional response, with significant upregulation of *ERG11*, *CDR1*, *CDR2*, *MDR1*, *MRR1*, and *TAC1B* genes (Figure 3D).

### 3.4. Epidermal Colonization Capacities of the Different C. auris Strains in the Mouse Skin Infection Model

**Figure 4:**
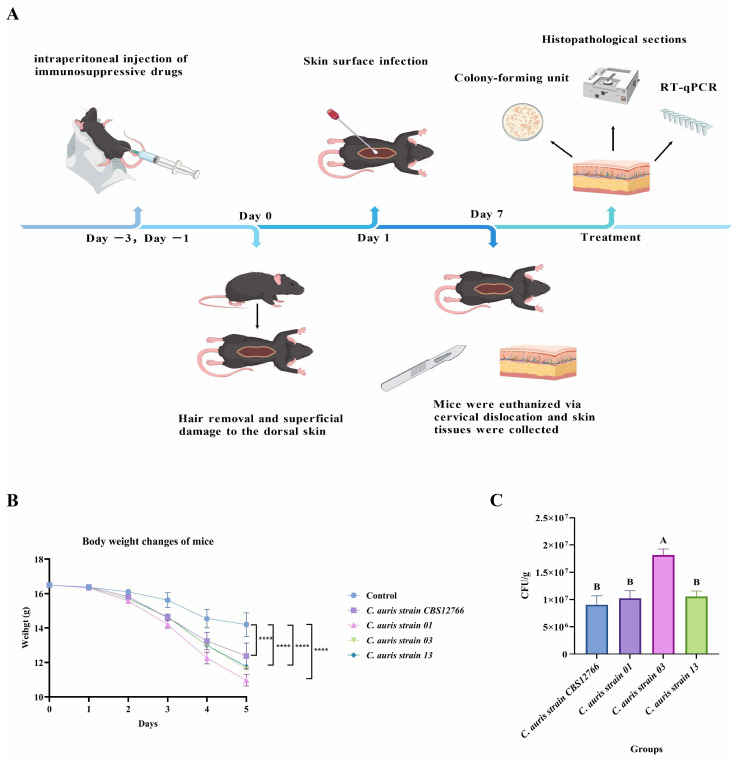
Mouse skin colonization model. (**A**) Flowchart of the mouse model establishment procedure. (**B**) Body weight changes in mice infected with four *Candida auris* strains. Data are presented as mean ± SD. Statistical significance was determined by two-way ANOVA followed by Tukey’s multiple-comparisons test. Statistical comparisons were performed using the data collected on day 5 post-infection. **** *p* < 0.0001. Control mice received phosphate-buffered saline (PBS). CBS2766, 01, 03, and 13 denote mice infected dorsally with the respective *C. auris* strains. (**C**) Colony-forming unit (CFU) bacterial load in the skin model. Statistical significance was determined by one-way ANOVA with Tukey’s multiple comparisons test. Different letters above bars indicate statistically significant differences (*p* < 0.05).

To assess the epidermal colonization capacity of the four *C. auris* strains in vivo, a murine skin infection model was established (Figure 4A). The body weight of the mice was monitored daily during the first 5 days post-infection. All infection groups exhibited varying degrees of weight loss over the observation period (Figure 4B); in contrast, the uninfected control groups showed minimal changes in body weight. Notably, mice in the *C. auris* strain 13 infected group displayed a near-linear decline throughout the monitoring period, while the remaining three infected groups also experienced significant weight loss, albeit to a lesser extent. These results indicate that infection with any of the four *C. auris* strains exerts distinct impacts on host health as reflected in body weight changes. CFU counts results revealed marked differences in colonization abilities among the four strains (Figure 4C). There was no significant difference in epidermal colonization ability among the *C. auris* strain CBS12766, *C. auris* strain 01, and *C. auris* strain 13, whereas the *C. auris* strain 03 exhibited significantly higher colonization capacity.

### 3.5. Different Host Immunity Responses to the Four C. auris Strains

**Figure 5:**
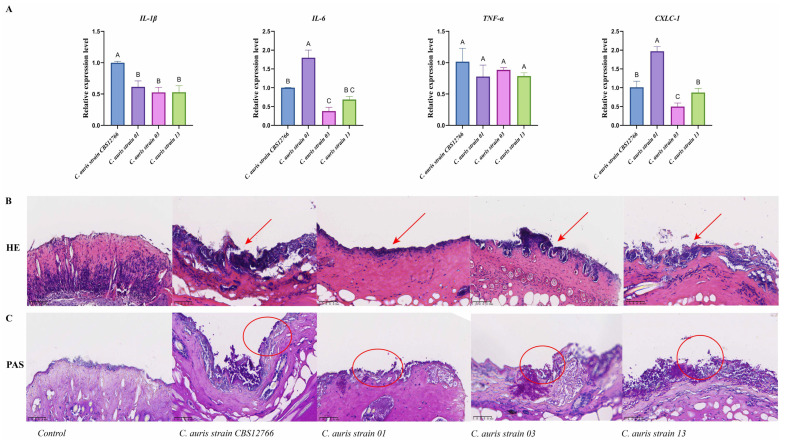
Relative expression of genes encoding inflammatory cytokines and chemokines, and histopathological features in a murine skin infection model. (**A**) Relative expression of inflammatory cytokines and chemokine genes in the murine infection model. Quantitative data are presented as mean ± SD. Statistical analyses for gene expression were performed using one-way ANOVA followed by Tukey’s multiple comparisons test. Different letters above bars indicate statistically significant differences (*p* < 0.05). (**B**) Hematoxylin and eosin (H&E) staining of infected murine skin sections. Inflammatory cell infiltration can be observed, as indicated by the arrows. (**C**) Periodic acid–Schiff (PAS) staining of infected murine skin sections. Fungal cells attached to the surface of the skin can be observed within the circles. In panels (**B**,**C**), the control group refers to mice treated with PBS as the control. Different letters (A–C) indicate statistically significant differences among groups (*p* < 0.05); identical letters indicate no significant difference.

During fungal skin infection, the expression levels of *IL-1β*, *IL-6*, *TNF-α*, and *CXCL-1* genes were markedly elevated, indicating a robust inflammatory response in the host. Among the four *C. auris* strains, infection with the *C. auris* strain CBS12766 induced significantly higher *IL-1β* gene expression compared with the other strains. In contrast, mice infected with the *C. auris* strain 01 showed no evident increase in *IL-1β* gene expression (Figure 5A), while *IL-6* and *CXCL-1* genes were more prominently upregulated. *TNF-α* gene expression levels were comparable across all infected groups. Histopathological analysis by H&E staining showed intact skin architecture in control mice, with a well-preserved epidermis and dermis, and no obvious inflammatory infiltration. In contrast, skin sections from *C. auris*-infected mice exhibited infection-associated pathological changes to varying degrees, including epidermal thickening, disturbed epidermal organization, and inflammatory cell infiltration primarily within the epidermis and superficial dermis (Figure 5B). Notably, the extent of inflammatory infiltration and tissue alteration differed significantly among the four infected groups representing the different strains, a finding consistent with their distinct cytokine induction profiles.

PAS staining further confirmed fungal colonization of murine skin following *C. auris* inoculation. No PAS-positive fungal elements were detected in the control group. In infected mice, however, magenta-stained PAS-positive structures consistent with fungal morphology were detected predominantly on the surface of the stratum corneum and within superficial epidermal layers (Figure 5C), supporting the observation of epidermal colonization. Across the four infection groups, PAS-positive signals showed subtle differences in distribution and abundance, suggesting strain-dependent variation in colonization behavior in vivo.

## 4. Discussion

*C. auris* has emerged as a formidable global health threat due to its rapid spread, high mortality rates, and broad spectrum of clinical manifestations [23,24]. Numerous studies worldwide have demonstrated that clinical isolates of *C. auris* exhibit exceptionally high rates of resistance to antifungals, with resistance to azole drugs being particularly prominent. Notably, resistance to fluconazole exceeds 90% in most geographic regions, severely limiting available therapeutic options in clinical practice [4,23]. Clinically, *C. auris* can cause superficial colonization of the skin and mucosa, bloodstream infections, and invasive systemic infections, including candidemia, endocarditis, and osteomyelitis [25]. Moreover, its epidemiological success is also linked to its environmental persistence, multidrug resistance, and the ability to form stable biofilms on medical surfaces, contributing to nosocomial transmission and recurrent infections [26,27]. In the current study, virulence traits and azole-resistance mechanisms investigations in clinical *C. auris* isolates revealed that strain-specific differences in adhesion, biofilm formation, and hydrolytic enzyme activity contribute to variations in virulence, making *C. auris* a complex pathogen requiring integrated phenotypic and molecular characterization [28].

Based on whole-genome sequencing, *Candida auris* can be classified into five major clades with distinct geographic distributions: Clade I (South Asian), Clade II (East Asian), Clade III (South African), Clade IV (South American), and Clade V (Iranian). In this study, *C. auris* strain CBS12766 was isolated from a blood sample in India, while three clinical isolates were obtained from patients in China. Molecular identification by Allele-Specific PCR assay [15] confirmed that all strains belonged to the South Asian clade (Clade I). Among virulence phenotypes, biofilm formation, adhesion, cell surface hydrophobicity, and phospholipase activity are critical determinants of fungal persistence and pathogenicity [29,30]. In the present study, *C. auris* strain 01 exhibited the strongest biofilm formation, while *C. auris* strain 03 and *C. auris* strain 13 had similar but significantly lower biofilm formation ability (Figure 1). Adhesion patterns mirrored biofilm formation, indicating that robust biofilm-forming strains adhered more effectively to host surfaces [31]. Phenotypic differences were observed among the strains. Notably, *C. auris* strain 01 displayed a different pattern. Although its fungal burden in skin tissue was relatively low, it caused the greatest body weight loss in infected mice and exhibited the highest level of azole resistance among the tested isolates. These observations suggest that skin colonization alone may not fully account for the differences in pathogenic behavior among strains. Rather, multiple factors, including antifungal resistance and other strain-specific phenotypic characteristics, are likely to influence the outcome of host–pathogen interactions. Further studies are needed to better understand how these traits contribute to the pathogenic potential of *C. auris* strains [32,33].

The antifungal drugs examined in this study encompass the principal therapeutic agents used for *Candida* infections. Their antifungal activities are mediated through distinct mechanisms, including inhibition of ergosterol synthesis, disruption of fungal membrane integrity, and interference with nucleic acid synthesis. Despite the emergence of multidrug-resistant *C. auris* strains worldwide, these compounds remain central to current antifungal treatment strategies [34]. Azole resistance in *C. auris* is multifactorial, involving alterations in drug targets, sterol biosynthesis, and efflux pump activity [7]. *ERG11*, encoding lanosterol 14α-demethylase, is the direct target of azoles, and mutations such as Y132F reduce drug binding and confer resistance [7,35]. Although the Y132F substitution in *ERG11* is widely regarded as a hotspot mutation associated with azole resistance in *C. auris*, our results demonstrated that Y132F alone was insufficient to confer an azole-resistant phenotype. Structural analyses showed that this substitution did not induce major conformational changes or alter the hydrogen-bonding network of *ERG11*, suggesting that its effect was limited to subtle modulation of azole–target interactions [11,12]. Accumulating evidence indicates that azole resistance in *C. auris* typically arises from the combined effects of *ERG11* mutations and additional resistance mechanisms, including increased *ERG11* expression and activation of efflux pump pathways mediated by transcriptional regulators such as *TAC1B* and *MRR1* [36,37]. In the absence of these cooperative mechanisms, Y132F is more likely to function as a permissive or contributory mutation rather than as a deterministic marker of azole resistance [38]. In addition, the *ERG3* gene contributes indirectly by preventing the accumulation of toxic sterol intermediates, often acting synergistically with *ERG11* mutations [39]. Efflux pumps, particularly the ABC transporters *CDR1* and *CDR2*, actively export azoles, while *MDR1* (MFS transporter) plays a minor role in *C. auris* compared to the situation in *Candida albicans* [11,36]. Furthermore, transcription factors, such as *TAC1b*, regulate *CDR1*/*CDR2* expression, and gain-of-function mutations enhance resistance, whereas *MRR1* impacts *MDR1* expression to a lesser extent [37]. Our results confirmed that fluconazole exposure upregulated *ERG11*, *CDR1*, *CDR2*, *TAC1b*, and *MDR1* expression in a strain-specific manner, reflecting diverse resistance mechanisms (Figure 3) among the strains.

Recent studies indicate that the epidemiological success of *C. auris* is linked to its adaptability to skin rather than to invasive pathogenicity alone [4,40]. Unlike *C. albicans*, *C. auris* readily forms stable skin colonization, acting as a persistent reservoir in hospitals, and systematically establishes a murine skin colonization model, demonstrating that *C. auris* can persist on skin and that skin barrier integrity and host immunity are critical [26]. In immunocompetent mice with intact skin, colonization and inflammatory responses are limited, making strain-dependent comparisons difficult [41]. In our study, by mildly disrupting the epidermal barrier and applying short-term cyclophosphamide immunosuppression, we achieved stable colonization and amplified local inflammation, enabling clear detection of strain-specific differences [2,42]. Moreover, in vivo, infection with different *C. auris* strains elicited distinct inflammatory responses (Figure 5). *IL-1β* and *TNF-α* acted as key pro-inflammatory mediators driving local inflammation [43]. In addition, *IL-6* contributed to the systemic acute-phase response, and *CXCL-1* promoted neutrophil recruitment to the infection site, enhancing local antifungal defense [28,44]. In our murine model, *C. auris* CBS12766 induced higher *IL-1β* expression, whereas expression levels of *IL-6* and *CXCL-1* genes were higher during infection by the *C. auris* strain 01 (Figure 5), while *TNF-α* expression was similar across the four strains. These findings highlight that phenotypic and molecular differences among the strains shape host immune responses in a strain-dependent manner [33,45].

Collectively, our study demonstrated substantial heterogeneity among the *C. auris* strains in virulence traits and azole resistance mechanisms. Differences in biofilm formation, adhesion, hydrophobicity, and phospholipase activity correlated with colonization capacity and inflammatory responses. Further analysis of azole resistance mechanisms confirmed that *ERG11* gene mutations and efflux pump regulation underpinned strain-specific azole resistance. These insights emphasize the importance of integrating phenotypic and molecular characterization in devising strategies for infection control and therapeutic intervention against the pathogenic fungus.

## Figures and Tables

**Table 1:** Antifungal susceptibility testing of the four strains.

Drugs	MIC (μg/mL)					
Strains	Fluconazole	Voriconazole	Itraconazole	Posaconazole	Amphotericin B	5-Fluorocytosine
*C. auris* strain 12766	>32	0.5	0.5	0.5	0.5	1
*C. auris* strain 01	>256	32	>64	>32	0.5	0.5
*C. auris* strain 03	4	1	1	0.5	1	1
*C. auris* strain 13	4	2	1	0.5	1	1

## Data Availability

The original contributions presented in this study are included in the article. Further inquiries can be directed to the corresponding authors.

## References

[1] Ettadili H., Vural C. (2024). Current global status of *Candida auris* an emerging multidrug-resistant fungal pathogen: Bibliometric analysis and network visualization. Braz. J. Microbiol..

[2] Towns K.A., Datta A., Thangamani S. (2024). Intradermal infection and dissemination of *Candida auris* in immunocompetent and immunocompromised mouse models. Microbiol. Spectr..

[3] Kim H.Y., Nguyen T.A., Kidd S., Chambers J., Alastruey-Izquierdo A., Shin J.H., Dao A., Forastiero A., Wahyuningsih R., Chakrabarti A. (2024). *Candida auris*-a systematic review to inform the world health organization fungal priority pathogens list. Med. Mycol..

[4] Chowdhary A., Sharma C., Meis J.F. (2017). *Candida auris*: A rapidly emerging cause of hospital-acquired multidrug-resistant fungal infections globally. PLoS Pathog..

[5] Hernando-Ortiz A., Mateo E., Perez-Rodriguez A., de Groot P.W.J., Quindós G., Eraso E. (2021). Virulence of *Candida auris* from different clinical origins in *Caenorhabditis elegans* and *Galleria mellonella* host models. Virulence.

[6] Ahmad S., Khan Z., Al-Sweih N., Alfouzan W., Joseph L. (2020). *Candida auris* in various hospitals across Kuwait and their susceptibility and molecular basis of resistance to antifungal drugs. Mycoses.

[7] Li Y., Hind C., Furner-Pardoe J., Sutton J.M., Rahman K.M. (2025). Understanding the mechanisms of resistance to azole antifungals in *Candida* species. JAC Antimicrob. Resist..

[8] Frías-De-León M.G., Hernández-Castro R., Vite-Garín T., Arenas R., Bonifaz A., Castañón-Olivares L., Acosta-Altamirano G., Martínez-Herrera E. (2020). Antifungal Resistance in *Candida auris*: Molecular Determinants. Antibiotics.

[9] Healey K.R., Kordalewska M., Jiménez Ortigosa C., Singh A., Berrío I., Chowdhary A., Perlin D.S. (2018). Limited *ERG11* Mutations Identified in Isolates of *Candida auris* Directly Contribute to Reduced Azole Susceptibility. Antimicrob. Agents Chemother..

[10] Wasi M., Khandelwal N.K., Moorhouse A.J., Nair R., Vishwakarma P., Bravo Ruiz G., Ross Z.K., Lorenz A., Rudramurthy S.M., Chakrabarti A. (2019). ABC Transporter Genes Show Upregulated Expression in Drug-Resistant Clinical Isolates of *Candida auris*: A Genome-Wide Characterization of ATP-Binding Cassette (ABC) Transporter Genes. Front. Microbiol..

[11] Li J., Coste A.T., Bachmann D., Sanglard D., Lamoth F. (2022). Deciphering the Mrr1/Mdr1 Pathway in Azole Resistance of *Candida auris*. Antimicrob. Agents Chemother..

[12] Li J., Coste A.T., Liechti M., Bachmann D., Sanglard D., Lamoth F. (2023). Novel *ERG11* and *TAC1b* mutations associated with azole resistance in *Candida auris*. Antimicrob. Agents Chemother..

[13] Eix E.F., Nett J.E. (2025). *Candida auris*: Epidemiology and Antifungal Strategy. Annu. Rev. Med..

[14] Xiao W., Zhou H., Huang J., Xin C., Zhang J., Wen H., Song Z. (2025). Comparative analyses of the biological characteristics, fluconazole resistance, and heat adaptation mechanisms of *Candida auris* and members of the *Candida haemulonii* complex. Appl. Environ. Microbiol..

[15] Carolus H., Jacobs S., Lobo Romero C., Deparis Q., Cuomo C.A., Meis J.F., Van Dijck P. (2021). Diagnostic Allele-Specific PCR for the Identification of *Candida auris* Clades. J. Fungi.

[16] Lei J., Xiao W., Zhang J., Liu F., Xin C., Zhou B., Chen W., Song Z. (2022). Antifungal activity of vitamin D_3_ against *Candida albicans* in vitro and in vivo. Microbiol. Res..

[17] Zhou H., Yang X., Zhou Q., Hu C., Xin C., Song Z. (2025). Character Virulence Association Factors and Gene Mutation Mediating Multidrug Resistance Phenotypes in *Candidozyma duobushaemulonii*. Mycopathologia.

[18] Datta A., Das D., Nett J.E., Vyas J.M., Lionakis M.S., Thangamani S. (2023). Differential skin immune responses in mice intradermally infected with *Candida auris* and *Candida albicans*. Microbiol. Spectr..

[19] Bai W., Wang Q., Deng Z., Li T., Xiao H., Wu Z. (2020). TRAF1 suppresses antifungal immunity through CXCL1-mediated neutrophil recruitment during *Candida albicans* intradermal infection. Cell Commun. Signal..

[20] Ebrahimi Barough R., Abastabar M., Moazeni M., Javidnia J., Valadan R., Bandegani A., Nosratabadi M., Haghani I., Spruijtenburg B., Armstrong-James D. (2025). Deciphering Fluconazole Resistance in *Candida auris* clade V: The Role of Efflux Pump Gene Expression and Ergosterol Pathway Mutations. Mycopathologia.

[21] Centers for Disease Control and Prevention (2023). Antifungal Susceptibility Testing for *Candida auris*. CDC Website. https://www.cdc.gov/candida-auris/hcp/laboratories/antifungal-susceptibility-testing.html.

[22] Sagatova A.A., Keniya M.V., Wilson R.K., Sabherwal M., Tyndall J.D., Monk B.C. (2016). Triazole resistance mediated by mutations of a conserved active site tyrosine in fungal lanosterol 14α-demethylase. Sci. Rep..

[23] Lionakis M.S., Chowdhary A. (2024). *Candida auris* Infections. N. Engl. J. Med..

[24] Ortiz-Roa C., Valderrama-Rios M.C., Sierra-Umaña S.F., Rodríguez J.Y., Muñetón-López G.A., Solórzano-Ramos C.A., Escandón P., Alvarez-Moreno C.A., Cortés J.A. (2023). Mortality Caused by *Candida auris* Bloodstream Infections in Comparison with Other *Candida* Species, a Multicentre Retrospective Cohort. J. Fungi.

[25] Schelenz S., Hagen F., Rhodes J.L., Abdolrasouli A., Chowdhary A., Hall A., Ryan L., Shackleton J., Trimlett R., Meis J.F. (2016). First hospital outbreak of the globally emerging *Candida auris* in a European hospital. Antimicrob. Resist. Infect. Control..

[26] Horton M.V., Nett J.E. (2020). *Candida auris* infection and biofilm formation: Going beyond the surface. Curr. Clin. Microbiol. Rep..

[27] Sansom S.E., Gussin G.M., Schoeny M., Singh R.D., Adil H., Bell P., Benson E.C., Bittencourt C.E., Black S., Guzman M.D.M.V. (2024). Rapid Environmental Contamination with *Candida auris* and Multidrug-Resistant Bacterial Pathogens Near Colonized Patients. Clin. Infect. Dis..

[28] Wang Y., Zou Y., Chen X., Li H., Yin Z., Zhang B., Xu Y., Zhang Y., Zhang R., Huang X. (2022). Innate immune responses against the fungal pathogen *Candida auris*. Nat. Commun..

[29] Ramage G., Saville S.P., Thomas D.P., López-Ribot J.L. (2025). *Candida* biofilms: An update. Eukaryot. Cell.

[30] Staniszewska M. (2020). Virulence Factors in *Candida* species. Curr. Protein Pept. Sci..

[31] Borman A.M., Szekely A., Johnson E.M. (2016). Comparative Pathogenicity of United Kingdom Isolates of the Emerging Pathogen *Candida auris* and Other Key Pathogenic Candida Species. mSphere.

[32] Bhargava A., Klamer K., Sharma M., Ortiz D., Saravolatz L. (2025). *Candida auris*: A Continuing Threat. Microorganisms.

[33] Phan-Canh T., Kuchler K. (2024). Do morphogenetic switching and intraspecies variation enhance virulence of *Candida auris*?. PLoS Pathog..

[34] Wang S., Pan J., Gu L., Wang W., Wei B., Zhang H., Chen J., Wang H. (2024). Review of treatment options for a multidrug-resistant fungus: *Candida auris*. Med. Mycol..

[35] Du H., Bing J., Hu T., Ennis C.L., Nobile C.J., Huang G. (2020). *Candida auris*: Epidemiology, biology, antifungal resistance, and virulence. PLoS Pathog..

[36] Rybak J.M., Doorley L.A., Nishimoto A.T., Barker K.S., Palmer G.E., Rogers P.D. (2019). Abrogation of Triazole Resistance upon Deletion of *CDR1* in a Clinical Isolate of *Candida auris*. Antimicrob. Agents Chemother..

[37] Rybak J.M., Muñoz J.F., Barker K.S., Parker J.E., Esquivel B.D., Berkow E.L., Lockhart S.R., Gade L., Palmer G.E., White T.C. (2020). Mutations in *TAC1B*: A Novel Genetic Determinant of Clinical Fluconazole Resistance in *Candida auris*. mBio.

[38] Rybak J.M., Sharma C., Doorley L.A., Barker K.S., Palmer G.E., Rogers P.D. (2021). Delineation of the Direct Contribution of *Candida auris ERG11* Mutations to Clinical Triazole Resistance. Microbiol. Spectr..

[39] Rybak J.M., Cuomo C.A., Rogers P.D. (2022). The molecular and genetic basis of antifungal resistance in the emerging fungal pathogen *Candida auris*. Curr. Opin. Microbiol..

[40] de Cássia Orlandi Sardi J., Silva D.R., Soares Mendes-Giannini M.J., Rosalen P.L. (2018). *Candida auris*: Epidemiology, risk factors, virulence, resistance, and therapeutic options. Microb. Pathog..

[41] Bruno M., Kersten S., Bain J.M., Jaeger M., Rosati D., Kruppa M.D., Lowman D.W., Rice P.J., Graves B., Ma Z. (2020). Transcriptional and functional insights into the host immune response against the emerging fungal pathogen *Candida auris*. Nat. Microbiol..

[42] Huang X., Hurabielle C., Drummond R.A., Bouladoux N., Desai J.V., Sim C.K., Belkaid Y., Lionakis M.S., Segre J.A. (2021). Murine model of colonization with fungal pathogen *Candida auris* to explore skin tropism, host risk factors and therapeutic strategies. Cell Host Microbe.

[43] Shyam Prasad Shetty B., Chaya S.K., Kumar V.S., Mahendra M., Jayaraj B.S., Lokesh K.S., Ganguly K., Mahesh P.A. (2021). Inflammatory Biomarkers Interleukin 1 Beta (IL-1β) and Tumour Necrosis Factor Alpha (TNF-α) Are Differentially Elevated in Tobacco Smoke Associated COPD and Biomass Smoke Associated COPD. Toxics.

[44] Burgess T.B., Condliffe A.M., Elks P.M. (2022). A Fun-Guide to Innate Immune Responses to Fungal Infections. J. Fungi.

[45] Santana D.J., Zhao G., O’Meara T.R. (2024). The many faces of *Candida auris*: Phenotypic and strain variation in an emerging pathogen. PLoS Pathog..

